# Using Implementation Mapping to develop protocols supporting the implementation of a state policy on screening children for Adverse Childhood Experiences in a system of health centers in inland Southern California

**DOI:** 10.3389/fpubh.2022.876769

**Published:** 2022-08-26

**Authors:** Mónica Pérez Jolles, María E. Fernández, Gabrielle Jacobs, Jessenia De Leon, Leslie Myrick, Gregory A. Aarons

**Affiliations:** ^1^ACCORDS Dissemination and Implementation Science Program, University of Colorado Anschutz Medical Campus, Aurora, CO, United States; ^2^Gehr Family Center for Health Systems Science and Innovation, Keck Medicine, University of Southern California, Los Angeles, CA, United States; ^3^Cecil G. Sheps Center for Health Services Research, University of North Carolina, Chapel Hill, NC, United States; ^4^Department of Health Promotion and Behavioral Sciences, Center for Health Promotion and Prevention Research, University of Texas Health Science Center at Houston School of Public Health, Houston, TX, United States; ^5^Suzanne Dworak Peck School of Social Work, University of Southern California, Los Angeles, CA, United States; ^6^Department of Psychiatry, University of California, San Diego, La Jolla, CA, United States; ^7^Altman Clinical and Translational Research Institute Dissemination and Implementation Science Center, University of California, San Diego, La Jolla, CA, United States; ^8^Child and Adolescent Services Research Center, San Diego, CA, United States

**Keywords:** Implementation Mapping, EPIS framework, federally qualified health center, ACEs screenings, PEARLS, toxic stress, trauma informed care

## Abstract

Adverse Childhood Experiences (ACEs) are defined as traumatic events occurring before age 18, such as maltreatment, life-threatening accidents, harsh migration experiences, or violence. Screening for ACEs includes asking questions about an individual's early exposure to these types of events. ACEs screenings have potential value in identifying children exposed to chronic and significant stress that produces elevated cortisol levels (i.e., toxic stress), and its associated physical and mental health conditions, such as heart disease, diabetes, depression, asthma, ADHD, anxiety, and substance dependence. However, ACEs screenings are seldom used in primary care settings. The Surgeon General of California has addressed this care gap by introducing ACEs Aware, an ACEs screening fee-for-service healthcare policy signed into law by Gov. Gavin Newsom. Since January 2020, Medi-Cal, California's Medicaid health care program, has reimbursed primary care providers for using the Pediatric ACEs and Related Life-events Screener (PEARLS) tool to screen children and adults for ACEs during wellness visits. To achieve the goals set by the ACEs Aware state policy, it is essential to develop and test implementation strategies that are informed by the values, priorities, and resources of clinical settings, healthcare professionals, and end-users. To address this need, we partnered with a system of federally qualified health centers in Southern California on a pilot study to facilitate the implementation of ACEs screenings in five community-based clinics. The health centers had broad ideas for an implementation strategy, as well as best practices to improve adoption of screenings, such as focusing on staff training to improve clinic workflow. This knowledge was incorporated into the development of an implementation strategy template, used at the outset of this study. We used the Exploration, Preparation, Implementation and Sustainment (EPIS) framework to guide the study and inform a participatory planning process called Implementation Mapping. In this paper, we describe how Implementation Mapping was used to engage diverse stakeholders and guide them through a systematic process that resulted in the development of the implementation strategy. We also detail how the EPIS framework informed each Implementation Mapping Task and provide recommendations for developing implementation strategies using EPIS and Implementation Mapping in health-care settings.

## Introduction

Adverse Childhood Experiences (ACEs) are defined as traumatic events occurring before age 18, such as maltreatment, neglect, life-threatening accidents, harsh migration experiences or exposure to violence (1). ACEs are pervasive, with 45% of children in the United States experiencing at least one ACE and 10% experiencing three or more ACEs, placing them at higher risk of negative physical and mental health outcomes (1). Addressing ACEs is critical to improving health equity, because these events are more prevalent among minority and immigrant communities due to exposure to poverty, discrimination, community violence, national disasters, and refugee experiences (2, 3). Screening for ACEs includes asking questions about an individual's early exposure to potentially traumatic events (4–6). Screening has the potential to facilitate a deeper understanding of the contributions of early experiences on an individual's developmental and health trajectory (4). The Surgeon General of the state of California has promoted the use of ACEs screenings in primary care by introducing an ACEs screening policy, called ACEs Aware, through the California Department of Health Care Services (7). This policy was funded through Proposition 56, which provides funding to improve health and increase interventions for youth. In January 2020, Medi-Cal, California's Medicaid health care program, began reimbursing primary care providers for using the Pediatric ACEs and Related Life-events Screener (PEARLS) tool to assess children and adults for ACEs during annual wellness visits (7). This state policy is unique in the country, as it promotes early identification of toxic stress, which is a prolonged physiological stress response that interferes with the brain, and its associated physical and mental health conditions, such as asthma, ADHD and anxiety, with the intention to connect these patients to needed services (8).

The ACEs Aware policy in California is a valuable pilot for the country. The economic and humanistic benefits of ACEs screenings remain debatable because it is important not only that screenings are completed in primary care settings, but that the information is used to engage families effectively with the goal of improving health. In order to be valuable, ACEs screenings must lead to timely, evidence-based interventions. Policymakers should consider how ACEs screenings are used, within a larger process of supporting families that have experienced traumatic events. Without the training necessary to implement trauma-informed care in healthcare settings, ACEs screening could re-traumatizing families; similarly, appropriate training is necessary for healthcare professionals to prevent compassion fatigue or burnout related to the process of discussing trauma with patients and caregivers on a daily basis.

The growing interest in ACEs screenings in primary care settings to address social determinants of health has been informed by research showing the benefits of this practice. Felitti et al. (9) stated that ACEs screenings can be therapeutic, as they allow the patient to reflect on the impact these experiences may have on their current health and to receive support from a health care professional. Identifying childhood adversity and offering appropriate interventions may ultimately decrease the risk of negative effects of ACEs, including problematic behavior and chronic illness in adulthood (10). Furthermore, screening may lead to earlier detection of patients who are at higher risk of mental and physical health challenges, prevent further ACEs among children, and present the opportunity to provide appropriate treatment (11–13). For example, Flynn and colleagues (13) conducted a systematic review of literature examining the use of trauma screening tools (e.g., Safe Environment for Every Child [SEEK; (14)] and Well Child Care, Evaluation, Community Resources, Advocacy, Referral, Education [WE CARE; (15)] in primary care settings and described four randomized controlled trials (RCTs) that found evidence of reduced risk of experiencing trauma and increased referrals to community resources. On the other hand, ACEs screening questions may cause discomfort for the patient and possibly disrupt health care relationships (4, 16). Additionally, we lack evidence as to whether increased ACEs screening efforts translate into better access to care for children (17). However, without effective implementation, reach, and sustainment of ACEs screenings, it will be difficult to determine the benefits of such screenings and any subsequent engagement in health services. Thus, there is a critical need for evidence regarding suitable strategies designed to support the successful implementation of ACEs screenings.

Rariden and colleagues (18) conducted a systematic review to explore the acceptability, feasibility, and implementation of ACE screenings across diverse settings (i.e., pediatric clinics, adult primary care, perinatal settings, patients' homes, and academic environments). The review found that most parents were willing to complete ACEs screenings on behalf of their children, and many parents were supportive of such practices. When exploring the feasibility of ACEs screenings, nine studies indicated that clinicians had concerns about adding time for screenings in already-busy visits, expressed lack of confidence about the implementation process, had uncertainty in processing past trauma with patients, and felt potential discomfort for families. Despite these concerns, however, there were no major disruptions reported after the implementation of screenings, and only one study identified an increase (<5 min) in the duration of the office visit. Rariden and colleagues (18) also found that training aimed at increasing clinician confidence, knowledge, and comfort with these screenings was associated with clinicians viewing ACEs screenings as acceptable and feasible. Other promising strategies included ensuring all staff participated in training (18, 19) and providing staff with adequate resources and multi-disciplinary support before the implementation (18–20).

To achieve the goals set by the ACEs Aware state policy, it is essential to develop and test implementation strategies informed by the values, priorities, needs and resources of clinical settings, professionals, and end-users (18–22). Implementation strategies refer to “methods to enhance the adoption, implementation, sustainment, and scale-up of an innovation.” [(23); p2] To address this need, we partnered with a large Federally Qualified Health Center (FQHC) with multiple locations in inland Southern California to engage in a two-year pilot study scaling up ACEs screenings in five community-based clinics. The FQHC partner had a broad idea of which implementation strategies and best practices might improve adoption of screenings, such as focusing on staff training to improving clinic workflow. This rich knowledge was complemented by information from the literature and by researchers' expertise (24). Yet, the implementation strategy at the outset of this study was lacking specific and comprehensive details necessary to effectively and confidently begin screening for ACEs. This study, funded by the National Institute of Mental Health, used the Exploration, Preparation, Implementation and Sustainment (EPIS) framework (25) to frame the project and to inform answers to questions posed using a collaborative process for planning implementation strategies called Implementation Mapping (IM) (26). IM is a systematic collaborative approach to develop and/or select and tailor multi-level implementation strategies. It uses a six-step iterative process that includes the explicit identification of all adopters and implementers, as well as a clear description of implementation outcomes, tasks, determinants, and change objectives. The process also includes delineation of the specific techniques (methods and practical applications of those methods) used to influence determinants and lead to implementation outcomes (26). EPIS is both a process and determinant framework that has been used in studies in widely varying healthcare systems, for different health conditions, and in multiple countries (27). The planning process started with the preliminary elements of an implementation strategy, and multiple collaborative mapping sessions were used to develop the details for each activity. The IM process was also used to tailor protocols to each participating clinic.

The purpose of this paper is to describe how the IM process and collaborations between the research team and diverse stakeholders representing healthcare leadership, clinic management, quality department, providers, staff, and caregivers contributed to the creation of a multi-faceted implementation strategy for ACEs screening implementation in five clinics. We report on the first four IM Tasks – Task 1: Conduct a needs and assets assessment and identify adopters and implementers; Task 2: Identify adoption and implementation outcomes, performance objectives, and determinants; Task 3: Identify and create implementation strategies; and Task 4: Produce implementation protocols and materials (26). We also describe how we used the EPIS framework and IM to guide the participatory process and plan implementation strategies. This process allowed the researchers and clinical health partners to collaboratively develop a detailed implementation strategy that reflected the nuanced and complex challenges of an FQHC operating during the COVID-19 pandemic.

## Methods

**Figure 1:**
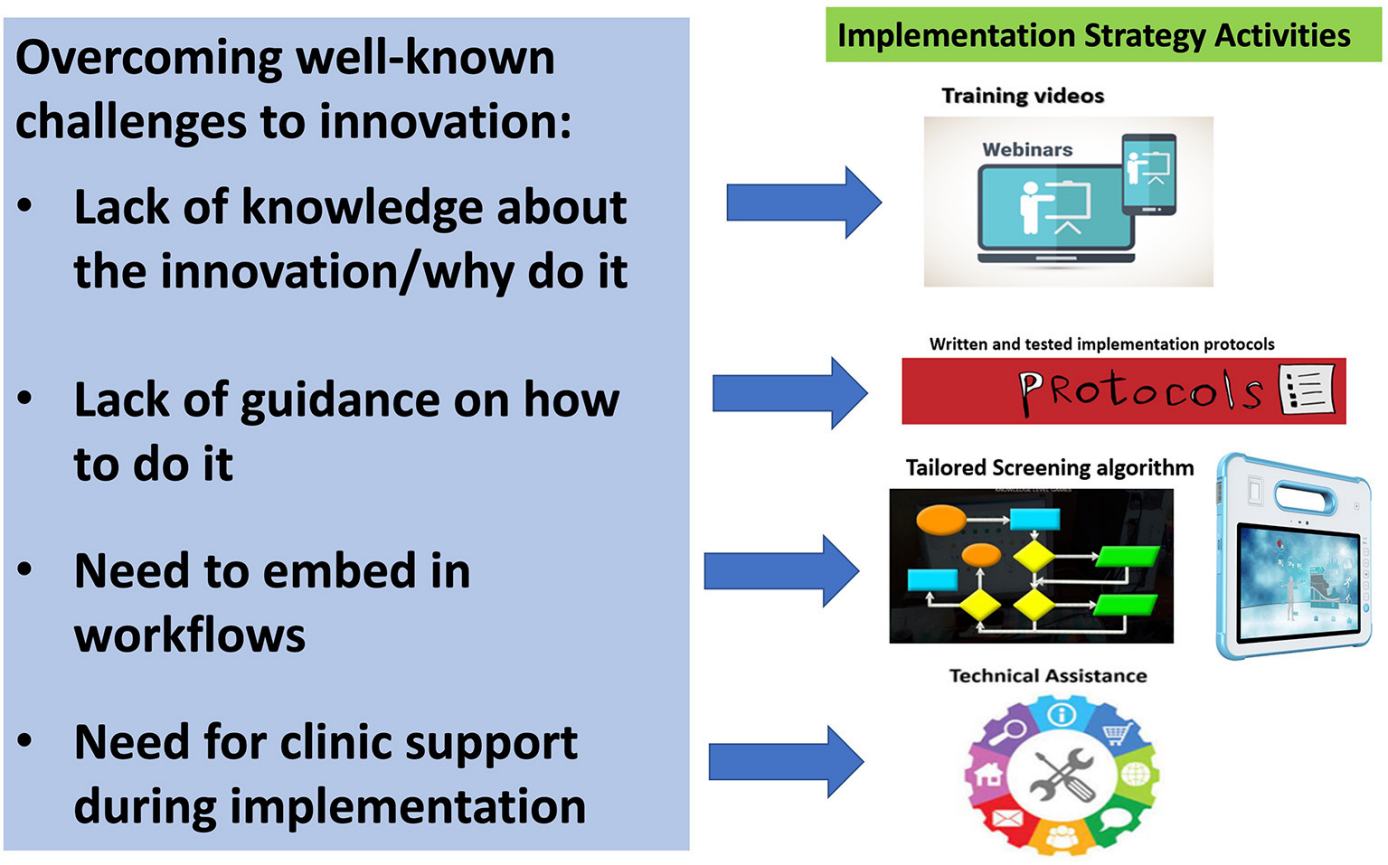
Template of the implementation strategy activities for ACEs screenings.

This study represents a partnership with five clinical sites that are part of a large FQHC system serving largely Hispanic/Latinx patients in frontier, rural, semi-urban, and urban regions in California. In late 2019, the partner healthcare system decided to adopt the ACEs Aware policy and reached out to the first author to support implementation efforts. An overarching implementation strategy template, designed to address identified challenges to implementing innovations in clinical settings and at the partner healthcare system (28–31), was co-created. As a sign of commitment to this effort, FQHC leadership gave approval for staff to devote the hours allotted to administrative duties to participate in implementation mapping activities and meetings. Figure 1 shows a slide used in planning meetings to introduce the strategy template with stakeholders. Conversations allowed for the expansion and development of the strategy with the use of EPIS and IM. The EPIS framework guided IM discussions for each of the phases [i.e., exploration, preparation, implementation, and sustainment; (32)]. In addition, this framework informed each IM task as related to the inner and outer contexts, the nature of the ACEs screenings as an innovation in the FQHC system, and bridging factors [i.e., formal arrangements and processes linking the outer system and the inner organization and clinic contexts; (32)].

The methods presented in this paper are novel in two ways. First, IM is a relatively new approach in terms of implementing practice change in community health centers to identify and/or design implementation strategies. In this case, IM was used to build on strategies that were identified during the development of the grant proposal, in which researchers collaborated with FQHC clinical partners (e.g., Director of the Research Department and Data Manager) and a Trauma-Informed Care (TIC) workgroup (which included the researchers) to develop a multi-faceted implementation strategy (33) to support ACEs screenings. The implementation strategies we selected (before beginning detailed planning using IM) included remote learning, use of technology to increase workflow efficiency during ACEs screenings, and technical assistance during implementation. Despite having these preliminary strategies, specific content still needed to be developed, and strategies needed to be re-considered and tailored to fit the realities of each of the five clinical sites. We used the IM process as a protocol to guide strategy development and planning. The EPIS framework helped us answer the various IM Tasks' questions. The framework also placed those questions in the implementation process, within the FQHC's inner organizational context, and within the outer policy context of the ACEs Aware initiative. This planning process guided participants to systematically co-design implementation protocols by specifying who had to do what to implement program components, identifying the needs related to increasing motivation and capacity, and tailoring strategies to improve implementation for each of the local clinical settings.

Second, this project is novel because we used the EPIS framework to provide the conceptual framework for researchers to consider the context in which the ACEs screenings were going to be implemented and to help address IM questions designed to guide planning efforts (e.g., who does what during each of the EPIS phases, what inner context organizational dynamics are at play, what are the considerations for individuals such as health care providers). In sum, IM provided a structure for planning the implementation strategies and the EPIS framework provided specific processes and constructs to help answer those questions. Both EPIS and IM informed group decision-making and identification of key determinants of change. This approach exemplifies how IM can be used with implementation frameworks to plan implementation strategies and advance the field of Implementation Science.

## Results

### Stakeholder engagement

**Figure 2:**
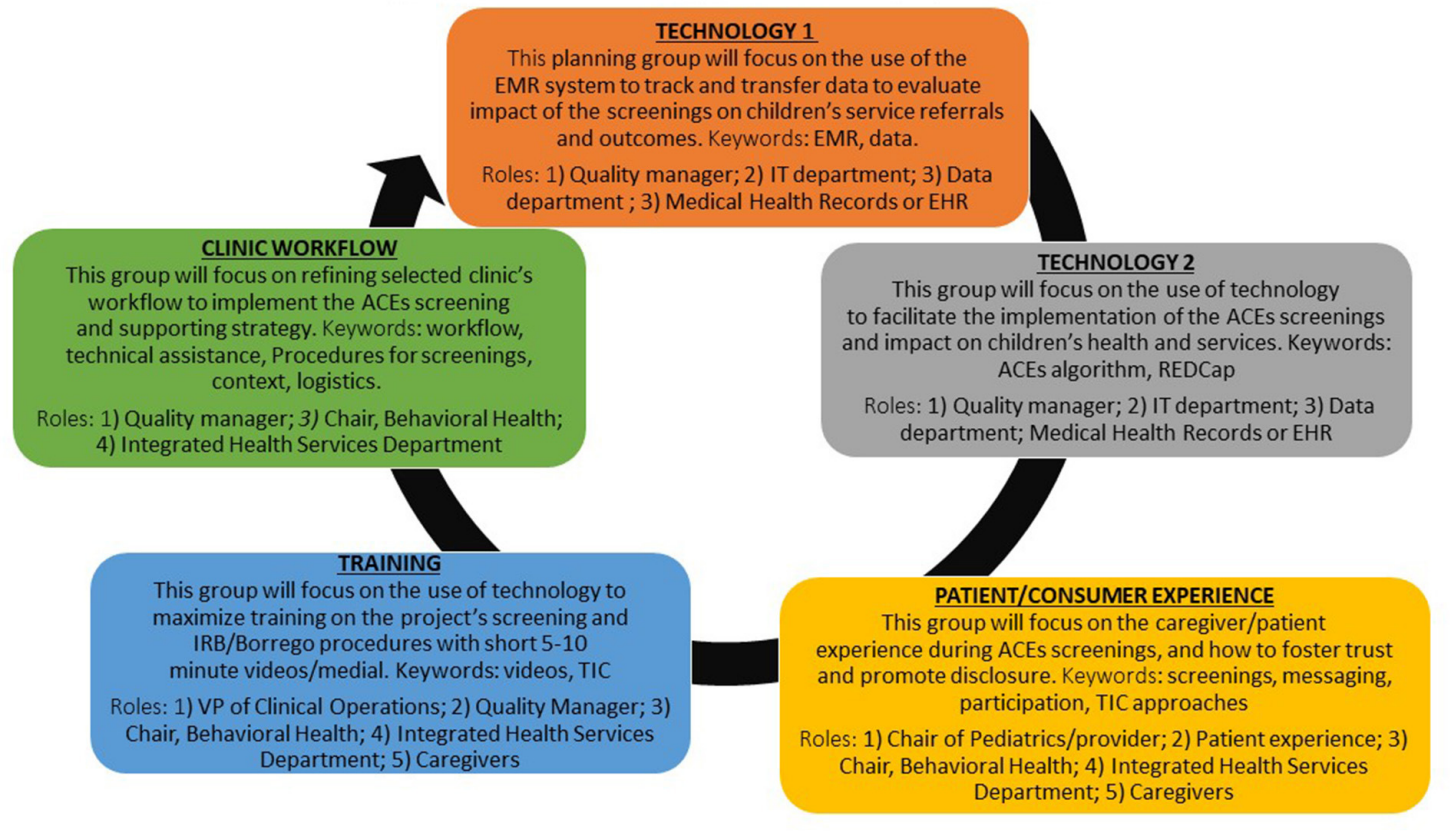
ACEs screenings planning-mapping sub-teams.

Central to the integration of IM and EPIS is engagement of stakeholders across all IM Tasks. The project started in May 2020 with an implementation team from the partner healthcare system: Director of Research, Data Manager, and Director of Pediatric Programs. Due to turnover during the COVID-19 pandemic in late 2020, the first two individuals left the organization. The Director of Pediatric Programs (DPP co-lead hereafter) remained, and a new data coordinator (data co-lead hereafter) joined the project. These two individuals are referred to as internal project co-leads, or champions. The initial implementation team was comprised of researchers, healthcare leadership and implementers, and end-users (i.e., caregivers of children ages 0–5 years). The team held two brainstorming sessions to identify initial stakeholders to be invited to the IM process based on the needs and characteristics of each of the implementation strategy activities. These individuals were identified based on their roles within the healthcare system and previous experience collaborating in various research projects with the first author since 2017. An email was sent to these 25 stakeholders, who represented key areas in the FQHC system that would support ACEs screenings and that were described in the previous section (i.e., technology transfer, use of technology, patient/caregiver experience, training, and workflow). Stakeholders were invited to an initial Zoom meeting, which was held 30 days after the study funding started. Based on this discussion, which touched on the specific IM tasks that would need to be accomplished throughout the project, attendees identified other colleagues whose expertise and enthusiasm for new programs would contribute to the planning and implementation process. Conversations in the initial meeting made it clear that stakeholders preferred to be involved in their area of expertise, and that administrative time was in short supply. As a result, stakeholders suggested the creation of subgroups based on selected strategy activities, and on areas of expertise/interest to improve the fit of the ACEs screenings for the participating clinics, and for FQHC system. Those areas included the use of technology to improve workflow, the transfer of data from EMR system for evaluation, training, caregiver (end-user) experience, and workflow (see Figure 2 for explanation of the goals set by the group for each mapping sub-team).

Changes to the composition of the sub-groups were made based on changes in the inner context (e.g., turnover), outer context (e.g., state mandate to isolate due to COVID-19 exposure and/or positive test), capacity to attend meetings and individual interest. Each group met two to three times throughout the IM process. This iterative process fostered the creation of tailored protocols to facilitate activities across the EPIS phases of preparation, implementation, and sustainment. Caregivers of pediatric patients provided feedback on ways to improve families' ACEs screening experiences. The IM planning meetings were structured to identify objectives and potential challenges, brainstorm ideas to overcome those challenges, and assign responsibilities to participants. Meetings with professionals were conducted in English, using the Microsoft Teams online platform, and each meeting was recorded and professionally transcribed to aid in analysis and identify ideas or tasks that would benefit from further discussion in later meetings or sub-groups. Meetings with caregivers were held mostly in Spanish on a conference phone call by the first author and a community health specialist. Due to the low quality of the call recordings with caregivers, two note takers were used to integrate and compare notes for accuracy. Caregivers received a gift card, delivered to their phones through text or *via* email, for their participation. The developed implementation protocols will inform the second phase of this study: a randomized stepped-wedge clinical trial to test the strategy in five clinical research sites.

### Characteristics of stakeholders involved

Consistent with the principals of IM, the planning process was carried out using a collaborative group process with a diverse group of stakeholders who shared responsibility for knowledge building and direction of the ACEs screening implementation. Forty-four stakeholders (77% female) participated in 12 IM meetings. The 52% (*n* = 23) of meeting attendees who provided demographic data reported their race or ethnicity as Hispanic (43%; *n* = 10), Middle Eastern (9%; *n* = 2), Asian (9%; *n* = 2), Black (4%; *n* = 1), and White (35%; *n* = 8). Professional roles included medical doctors, clinic managers, medical assistants, medical scribes, nurses, and technology managers. Separately, we included a group of end-users (13 caregivers), who provided feedback on the screening process. All caregivers identified their ethnicity as Hispanic and their gender as female; the average age was 27 years old. Just over half of the caregivers preferred to participate in the IM conversations in Spanish, rather than English.

### The EPIS framework informed the implementation mapping process

**Figure 3:**
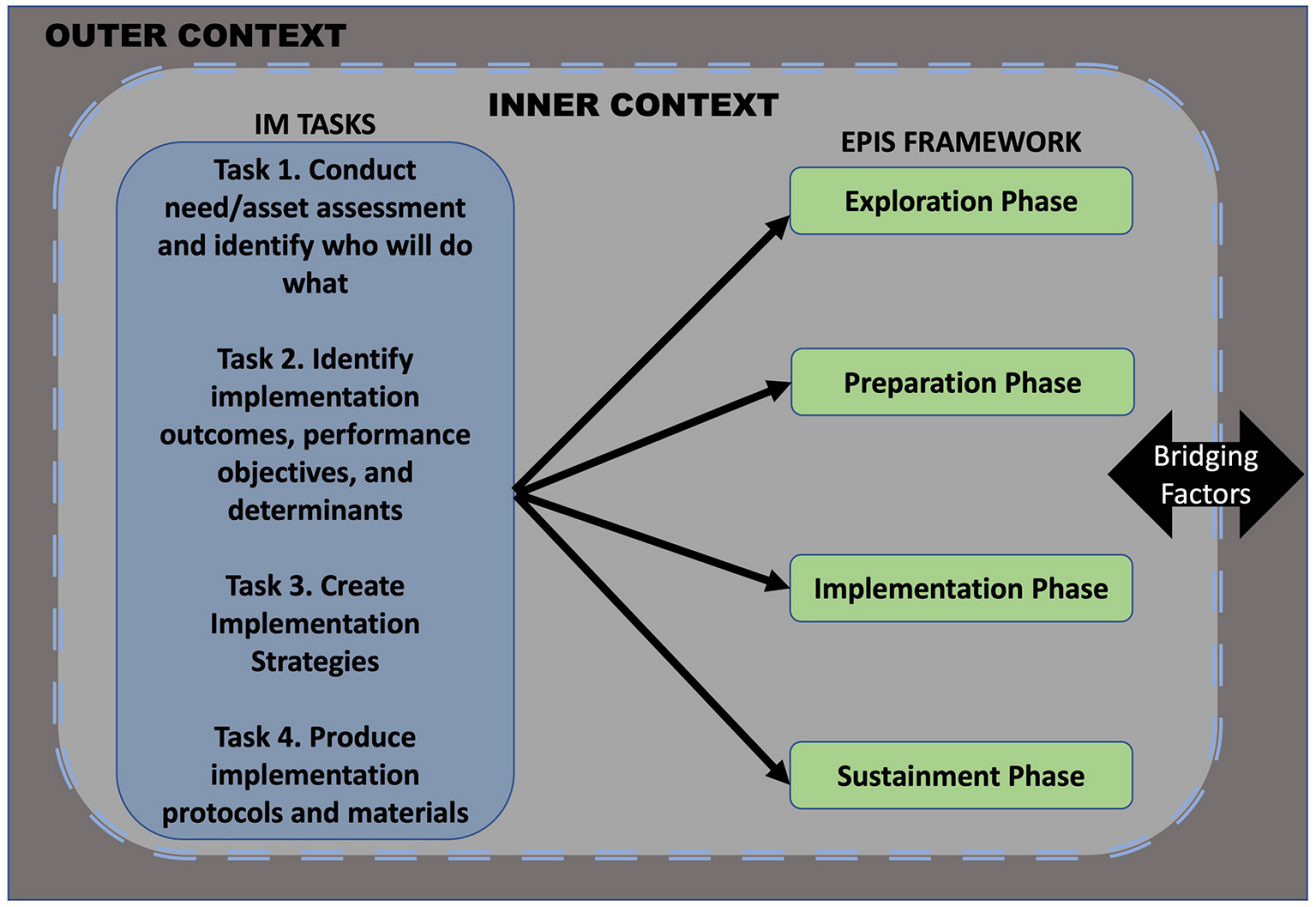
EPIS-informed implementation mapping process.

We considered each phase of the EPIS framework during each IM task. This helped ensure that we would have strategies that would be appropriate for the various phases of EPIS, from Exploration through Sustainment. We also considered the inner context of the organization and clinics, the outer system and community context, and bridging factors that link outer and inner contexts (e.g., funding, policies, and characteristics of the ACEs screenings when identifying the most salient factors influencing implementation and making decisions across the IM strategy planning steps) (30, 31) (Figure 3).

This approach allowed us to account for the dynamic nature of the healthcare system due to inner and outer context characteristics and events in general and during the COVID-19 pandemic in particular, the nature of the ACEs screenings (i.e., benefits vs. burdens), and the need to approach planning through a lens of equity and inclusion (32). The main IM strategy development activities lasted seven months, with meetings of 40–60 min. Meetings were facilitated by the first author, second author, and by the DPP co-lead.

### IM task 1: Conduct a needs and assets assessment and identify adopters and implementers

In 2020, the TIC workgroup conducted anonymous organizational surveys to assess training needs among service providers, awareness of the ACEs Aware policy, perceived ability to successfully screen for ACEs in their clinic after taking the state training, leadership support, and workforce morale. The survey was open online from 17 July to 4 August 2020, and a total of 162 individuals were invited to participate, with 52 individuals completing the survey (36% response rate). Of those, 32 (61%) were clinical providers (MDs and DOs), 17 (33%) were nurse practitioners, and 3 (6%) did not report. More than half of survey participants found the ACEs training relevant (52%) to their clinical practice, and most (74%) said they had the training and information needed to screen patients based on completing the state's required 2-h ACEs Aware training. Some participants were not clear on how the workflow would accommodate this new screening and suggested including nurses, medical assistants and case managers in the screening process and training. Results from a separate leadership survey conducted in early 2021 identified internal factors related to the partner healthcare system that could challenge the implementation of the ACEs screenings. Those factors included high levels of burnout at the FQHC and a need for leadership to improve self-care among employees and promote TIC across the organization.

#### EPIS framework contributions

The exploration and preparation phases of the EPIS framework informed this task by providing additional guidance on what to consider when examining needs, assets, and challenges based on organizational characteristics (i.e., inner context) and their potential impact or fit on the implementation phase (see Table 1). The mapping sub-teams discussed these areas based on the outer (i.e., state ACEs Aware state policy), and inner (i.e., turnover and personnel reorganization) context characteristics as well as bridging factors (33). That mutual interdependence is seen in the state requirement to complete a 2-h online training for clinic personnel involved in conducting screenings and the submission of ACEs screenings scores, and the ability of clinics to submit billing codes to the state for financial reimbursement (i.e., $29 for each completed ACEs screening, once a year for each patient).

**Table 1:** Implementation Mapping: task 1.

**Inner Context**	**Organizational characteristics**	**How it will impact implementation?**	**Who can do something about it?**
Leadership	Hierarchical structure of the organization	Communication flows from the top down, which takes longer	Leadership: Behavioral Health Department Chair, Pediatrics Chair, Chief Clinical Officer
	Leadership at the organization as a whole not very integrated with leadership in the field (clinics)	Time needed for upper leadership to check in with clinic leaders and vice versa	
	Shift from a centralized system and into allowing more independence to decision-making at the clinical level	Take longer for access to clinics for planning	
Capacity	Severely diminished due to COVID-19 pandemic Research department dismantled and closed Floating/admin personnel reduced to a minimum Financial crisis due to COVID-19 impact Extreme turnover	Delayed start time for screenings Loss of implementation team members Less time for implementation Shrinking workforce; less time for training or administrative activities Lack of implementers; requires new team members to be introduced to project	Clinic Managers; Project Co-Lead/Director of Pediatric Practice* (DPP); Trauma-Informed Care (TIC)** Workgroup; Community Health Advisors
Organizational Structure/ Culture	Remote work and big size organization	Makes planning longer and through multiple groups/reliance on Microsoft Teams and zoom	Project co-lead/ champions
	Organizational re-structuring, new roles, layoffs, turnover, uncertainty, external monitoring; at the provider level, staff burnout, change fatigue, lack of staff understanding and little education about changes	Burnout and fatigue regarding innovate; role confusion	Leadership: Behavioral Health Department, Pediatrics, Chief Clinical Officer
	Co-Leads representing operations and data	Director of Pediatric Practice (DPP) and Data Coordinator (Data Co-Lead)	
General Mapping Group	Need to inform and educate patients about toxic stress, ACEs***, and the impact on their health outcomes.	Lower buy-in and engagement	TIC Workgroup
	Lack of trauma-informed care (TIC) awareness	Lower buy-in and engagement	TIC Workgroup
Workflow Mapping Group	Lack of staff at the clinics to champion/implement	Low readiness for change and few resources in place for implementation	Clinic managers; Leadership
	Competing demands for implementers' attention	Lower buy-in and engagement	Clinic managers
	Change fatigue and burn out	Lower buy-in and engagement	Leadership; Project co-leads/ champions; Clinic managers
			Pediatricians
	Lack of appropriate training and clarity on who is doing what, when, how; Confusion on what to do with caregiver declines and deviation from plans	Low readiness for change and resources in place for implementation	Academic partners Project Co-leads
	Not enough time to prepare for implementation (2 weeks or less)	Low readiness for change and resources in place for implementation	Clinic managers; Research Team; project co-lead
	Need to improve efficiency of workflows	Low fidelity and sustainment	Project Co-leads; Clinic Managers; Research Team
	Instructions are complicated – too many arrows to follow to know what to do	Low buy-in and sustainment	
Technology Mapping Groups	Lack of leveraging technology to improve efficiency	Low fidelity and sustainment	Academic partners; Project Co-leads
	Use of USC tablets too complicated	Low fidelity and sustainment	
	Need to ensure consistent data entry – who is doing what, what is working, deviation from plans – that is necessary for refinement	Fidelity	Project co-lead (EHR systems and dashboard)
Leadership Group; All Mapping Groups	Lack of personnel due to COVID-19 vaccine policy in California	Low readiness for change and lack of resources in place for implementation	ACEs Aware Leadership; Project Co-Lead/DPP; Clinic Managers; Research Team
Patient/ caregiver experience Mapping Group	Low reading levels from caregivers	Low disclosure; lower buy-in and engagement	ACEs Aware Leadership; Project Co-leads; Clinic managers; Research Team
	Patients not disclosing / refusing to complete forms	Lower public health impact; policy not meeting its goals	
	Lack of resources in place for referrals after screenings	Low buy-in and sustainment	
	Caregivers not knowing anything about the new program in advance; takes significant time to educate caregivers	Lower buy-in and engagement; Lack of trust in providers/clinic	
Leadership Group; All Mapping Groups	Lack of personnel due to COVID and Vaccine policy in California	Low readiness for change and resources in place for implementation	
**Outer context**	**Organizational characteristics**	**How it will impact implementation?**	**Who can do something about it?**
	Ongoing changes to the ACEs Aware policy in terms of procedures, expectations, tools	Creates confusion; requires ongoing feedback loops of rapid assessments	PEARLS Developers; ACEs Aware Leadership; CALQIC**** Leadership
	Scripts for implementers to use made available in October 2021 (policy started reimbursing clinics in January 2020)	Creates confusion; requires ongoing feedback loops of rapid assessments	
	No direct communication between ACEs Aware leadership and Health leadership	Gaps in knowledge; lack of up-to-date information; lower fidelity to state guidelines	Project DPP* Co-Lead has indirect communication through CALQIC*** and can serve as liaison
	**Innovation characteristics**	**How it will impact implementation?**	**Who can do something about it?**
	Innovation is attached to state reimbursement (i.e., relative advantage)	Strong incentive to adopt the innovation and do what is needed to obtain reimbursement; additional procedures not attached to reimbursement may not be prioritized	Project co-leads/ champions; EHR systems co-lead
	Addresses a key need identified in the patient population for this FQHC system: trauma	Increased fit of the ACEs screenings with the FQHC mission and goals	Leadership; Project DPP* co-lead; Clinic managers; Pediatricians
	Visibility through service grants from the state; free training and access to resources	Learning from the community informs this pilot's efforts; shared lessons learned; access to policymakers	PEARLS Developers at ^∧^UCSF; ***CALQIC

Leadership: Behavioral Health Department Chair, Pediatrics Chair, Chief Clinical Officer; *DPP, Director of Pediatric Practice; **TIC, Trauma-Informed Care; ***ACEs, Adverse Childhood Experiences; ****CALQIC, California ACEs Learning and Quality Improvement Collaborative – State funded service grant; ^∧^UCSF, University of California San Francisco.

This preliminary information informed the priorities for future planning, such as discussions about who would be leading and conducting the ACEs screenings. This was critical given the high staff turnover and shortages in clinic personnel at the time of these discussions because of the COVID-19 pandemic. With that agenda in mind, the first planning meeting was held using Zoom, for 60-min, with all stakeholders involved to introduce the new ACEs screenings initiative. The agenda included a description of the broad implementation strategy proposed in the funding proposal and the logic model behind it. (34) We followed IM guidelines to identify not only the barriers to implementation (Task 1) but also to consider the identification of specific implementation actions (i.e., performance objectives) and the determinants likely to influence them. To accomplish this, we asked the following questions: “Why do you think [name of the healthcare system] has decided to adopt this state policy?” “Who will make the resources needed to support the screenings?” and “Who can champion these screenings at each clinic?” The group then discussed how they would like to organize themselves to tackle each implementation activity and further develop the details on “who, what, how and when.” These discussions allowed the groups to identify “Who will do what?” as well as potential gaps in key stakeholder involvement, such as a need for outreach to leadership (e.g., Chief Medical Officer, Director of Pediatrics Department, and Director of Adult Services) to provide needed resources and to collaborate on problem-solving. As a result, the first and third authors, and the DPP co-lead convened bi-monthly Zoom meetings with leadership starting early on during the EPIS exploration and preparation phases for planning processes. These meetings will continue throughout the duration of the study.

One example of the benefits of including end-users during the IM process, and early on during the EPIS preparation phase, was the fact that caregivers who participated in our project shared a need to add strength-based questions to the ACEs screenings to showcase families' resilience. It was also deemed important to clarify that all caregivers of children ages 0–5 years were being asked the ACEs questions to avoid caregivers feeling singled out. The team added these strategies to the implementation protocol with the goal of improving families' experiences during ACEs screenings in primary care settings, and to address potential unintended consequences such as further stigmatization.

### IM task 2: State adoption and implementation outcomes, performance objectives, and determinants; create matrices of change

Given the barriers and opportunities that had been identified in Task 1, the team continued to describe targets for change and desired outcomes. For this Task, the research team shared with stakeholders the original implementation strategy template and broadly defined intended outcomes (i.e., reach, acceptability, and feasibility of the implementation strategy activities) as a starting point for stakeholder discussions. The IM process allowed the team to refine the template by identifying concrete performance objectives (implementation sub-tasks/behaviors) that would lead to those outcomes and to confirm with stakeholders that those intended outcomes were relevant and valued. One example of this feedback was that stakeholders identified a need to support efficient workflows and clinical care team procedures during the planning and implementation of the ACEs screenings, to increase likelihood of sustainment. The overall goal of this step was to focus on identifying the appropriate “implementers” and concrete activities (or Implementation Tasks) for them to overcome key challenges identified during the needs assessment (Task 1; i.e., high turnover, financial stress, inefficient workflows). The performance objectives were framed in terms of specific Tasks and who would complete the Tasks to integrate ACEs screenings into existing organizational and clinic workflows and procedures. Identifying performance objectives for implementation and sustainment through the use of several IM Matrices of Change allowed us to identify key determinants (e.g., knowledge) for each specific performance objective. In this project, we organized the activities in this step according to EPIS phases.

#### EPIS framework contributions

The performance objectives and outcome discussions during this Task were integrated into a table framed around each of the phases of the EPIS framework (e.g., Who will be responsible for the identified objectives and outcomes during the preparation of ACEs screenings? During their implementation at the five clinical settings? During sustainment?) These questions were asked based on the inner and outer context characteristics of the FQHC system. Even though it was at times difficult for stakeholders to plan too much ahead (e.g., sustainment phase), they appreciated the systematic and sequential approach of this step. See Table 2 for a summary of this step's products.

**Table 2:** Implementation Mapping: table of performance objectives by EPIS stage and constructs.

	**Responsible person**	**Performance objectives**	**Awareness and perceptions of ACEs screenings and implementation Activities**	**Implementation outcomes**
	**Preparation**			
**Inner context**	Leadership	PO1. Troubleshoot and remove obstacles related to the new heath initiative PO2. Support employees' efforts to implement screenings, improve caregiver disclosure. and participate in study PO3. Facilitate Trauma-Informed Care (TIC) Training for clinics and advisory group	AP1. Troubleshoot and acknowledge availability of staff AP2. Prioritizes needs for this project AP3. Feel positive about overcoming barriers and maintaining quality	Use Task 1 assessment of challenges to develop a plan to integrate ACEs screenings into clinic's workflows and procedures
	Research team and DPP co-lead	PO1. Gain support from care team at each clinic for the ACEs screening and research study PO2. Increase awareness about TIC at each clinic PO3. Creates resource sheets for caregivers for support services and behavioral health referrals PO4. Convey support for clinic personnel during implementation PO5. Establishes clear standards for implementation	AP1. Describe ACEs screenings/TIC care as an improvement over usual care to ID toxic stress AP2. Perceive the academic-clinical partnership as contributing to the healthcare system mission and goals	
	Data Coordinator (Data Co-Lead)	PO1. Set up the data tracking system for the five new clinics using Tableau PO2. Set up coding and billing system for state reimbursement for the five new clinics	AP1. Clinics perceive the data tracking and billing process as easy to follow/already set up AP2. Screenings are embedded into each clinic's workflow and in an efficient manner	
	Clinic Managers	PO1. Agree to participate in the implementation effort for ACEs screenings PO2. Allow clinic care team to be part of workflow planning and training	AP1. Be inclusive AP2. Care teams perceive as knowing how to successfully screen (efficacy)	
	Information Technology Manager	PO1. Be available for questions on how to access REDCap from clinic tablets; ensure Wi-Fi access PO2. Make sure the PDF printing feature is active for screeners to print PDFs from REDCap system	AP1. Perceive the use of technology in ACEs screenings as part of clinics' screenings services	
	Training Department	PO1. Review training materials and provide feedback based on their expertise leading training efforts in the healthcare system		
**Outer context**	Research Team and DPP co-lead	PO1. Reach out to ACEs Aware state policy makers and related state websites to stay abreast of changes to the ACEs Aware policy PO2. Reach out to ACEs screening tool developers (sub-contracted by the state) to share concerns from researchers, caregivers and clinic personnel and offer feedback for improvement to increase the cultural appropriateness	AP1. Clinic personnel perceive that they are abreast of ACEs Aware requirements, and that they are addressing unintended consequences and a need for cultural lens when implementing ACEs screenings	
		PO3. Add a culturally appropriate TIC training by hiring a national organization to train care teams at each clinic		
	**Implementation**			
**Inner context**	Medical assistants	PO1. Attend ACEs screening and research procedures training	AP1. Knowledge / remote learning	Implementation of the ACEs screenings and strategy activities with fidelity and documenting adaptations
		PO2. Follow procedures before, during and after screenings	AP2. Perceived guidelines for research / consenting	
		PO3. Document to submit billing for state re-imbursement		
	Community Health Advisors	PO1. Communicate with Medical Assistants and substitutes on screenings when clinic is short-staffed	AP1. Experience with CALQIC program	
		PO2. Provide resources to caregivers and follow up after screenings		
	Clinic Managers	PO1. Identify eligible children every week	AP1. Acknowledge and arrange for availability of screeners	
		PO2. Supervise completion of screenings (5 per week)		
	DPP Co-Lead	PO1. Motivates clinic staff to participate in study surveys and interviews	AP1. Experience with state-funded California ACEs Learning and Quality Improvement Collaborative (CALQIC)	
		PO2. Schedules a visit to the clinic for coaching and follows up with consultation call (every 10 weeks)		
	**Sustainment**			
	Leadership	PO1. Distribute study results within the healthcare system, and to board of directors and state	AP1. Experience disseminating research across the organization.	ACEs screenings and strategy activities are scaled up to other clinics and become part of primary care visit practices
			AP2. Existing relationships with state policy makers.	

Leadership: Behavioral Health Department Chair, Pediatrics Chair, Chief Clinical Officer; REDCap: (Research Electronic ***Data*** Capture) is a browser-based, metadata-driven EDC software and workflow methodology for designing clinical databases.

During this Task, having the voices of professional stakeholders with diverse backgrounds as well as the voices of caregivers allowed for sometimes difficult but needed conversations about the balance between the potential benefit of ACEs screenings [e.g., families perceiving the ACEs screenings as a preventive tool (Vides, B, oral communication, 7 January 2022)] and potential unintended consequences. Those potential consequences included stigmatization, given the high prevalence of ACEs among US youth, and among minority communities (1–3), and increasing discomfort and mistrust with caregivers as a result of being asked ACEs questions during a primary care visit. More specifically, caregivers shared that the questions in the PEARLS screening tool were too direct and feared that because of mandated reporting, families could become involved with child protective services and potentially separated as a result of answering the questions.

Actions to address these concerns included adding two strength-based questions to the ACEs screenings; informing caregivers in advance that these screenings were happening as “usual care” at their clinic; providing a comprehensive introduction to the ACEs screenings that explained that all caregivers were being asked these questions to avoid caregivers feeling singled out; explaining that the screenings were voluntary; and having concrete resources and services available to support caregivers after the screenings were completed, based on the child's needs. Champions were identified to carry out suggestions to overcome these concerns as reflected in Table 2. In addition, stakeholders were concerned about children who are deemed at intermediate or high-risk levels for toxic stress (based on ACEs screenings and state guidance on scoring thresholds), and in need of linkage to support services, not having access to supports due to lack of services in some of communities. As a result, the research team in collaboration with project co-leads and Community Health Advisors co-developed a centralized database using Excel with a list of family support services (including mental health services), organized by each of the clinics' counties. The database was updated bi-weekly by the PhD student, who called the main services mapped in the database to ask about estimated waiting time for patients at the time of the call. She also asked about agency closures, as well as the agencies' awareness of the ACEs Aware state policy. This database was shared with the referral specialist and Community Health Advisor at each participating clinic to support pediatricians' efforts to link families to services after ACEs screenings.

One example of the benefits of this participatory and co-creation planning process became clear when the two initial implementation champions at the partner healthcare system (i.e., Director of Research and Data Manager) left the organization within the first 2 months of the study. Instead of causing a major disruption to the IM process, there was a relatively smooth transition, which was likely due to clearly articulated goals and planning processes. The Director of Pediatric Programs or DPP stepped in to assume a leadership role as a co-lead, and a new data manager project co-lead was promptly identified because these two individuals had participated in Task 1 of the IM process.

### IM task 3 and 4: Choose change methods and develop practical applications for program use; produce implementation protocols and materials

Given the dynamic nature and inter-dependence across the five mapping sub-groups, we are reporting the main activities of the last two Tasks together. The mapping sub-groups started by reviewing the list of factors that could serve as barriers to the ACEs screenings and strategy activities and by adding new stakeholders (e.g., caregivers during the preparation phase) and selecting the determinants that were a priority for the groups. These conversations informed the final linkage of who was doing what (agents), their performance objectives, relevant determinants of success, change methods, and practical applications in clinical settings and at the healthcare organizational levels. These linkages were built to expand and refine the implementation protocol for program use that was initiated in Task 2.

#### EPIS framework contributions

Given the characteristics of the ACEs innovation involving a pediatric screening procedure that requires coordinated actions from multiple implementers (e.g., clinic managers, medical assistants, pediatricians, and community health workers), and within a dynamic organizational setting, we focused on inner context areas such as workflow, training, information technology, and electronic healthcare records systems. See Table 3 for a table mapping the sequence of activities and tailored practical applications and materials for the implementation protocols. Identification of effective leadership was included in the IM process, because the EPIS framework highlights this as a key factor in successful implementation of innovations. During the IM process of identifying performance objectives, the team discussed what leaders and champions can do to support implementation during all four EPIS phases and rationale for leadership support at multiple system and organization levels (35). This is an example of how frameworks can inform performance objectives and methods of change.

**Table 3:** Implementation Mapping: Steps 3 and 4.

**Preparation phase**				
**Outcome: Develop a plan to integrate ACEs screenings into clinics' workflows and procedures**				
**Agent**	**Performance objectives**	**Determinants (why would they do these things?)**	**Change methods**	**Practical applications and materials**
Leadership	PO1. Remove obstacles related to ACEs screenings and study procedures activities PO2. Support employees' efforts to implement screenings, improve disclosure from caregivers and participate in study PO3. Facilitate Trauma Informed Training for clinics and advisory group	Perceived added value to care/ improved care Perceived expectations / norms	Information transfer Persuasive communication through providing added care value	Quarterly meetings with academic partners and DPP Memo emailed to clinics endorsing the projects
DPP Co-lead	PO1. Gain support from care team at each clinic for the ACEs screening and research study PO2. Increase awareness about TIC at each clinic	Previous experience with CALQIC	Persuasive communication	Power point slides and discussion points in webinars; Provide evidence of success of the ACEs screenings already in place at two other clinics since 2020
		Time		
		Familiarity		
Data Co-lead	PO1. Set up the data tracking system for the new five clinics using Tableau PO2. Set up coding and billing system for state reimbursement for the new five clinics	Time Expertise with data and billing systems for all programs at the organization	Skill building Modeling Persuasion	Dashboard system created for ACEs screenings data entry and retrieval (i.e., Tableau)
Clinic Managers	PO1. Agree to participate in the study PO2. Allow clinic care team to be part of workflow planning and training	Leadership support Time	Monitoring and feedback Facilitation	Emails and communications during staff meetings
Information Technology Manager	PO1. Agree to be contact person for technical problems with the iPad Tablets for screenings	Expertise in use of iPad Tablets in primary care	Information transfer Skill building Technical assistance/capacity building	Emails Phone number
Training Department	PO1. Lead future ACEs screening training efforts at the organization level	Expertise in leading personnel trainings	Facilitation Organizational planning	Training manual reviewed by this team and materials branded with the organization's logos, templates
**Implementation Phase**				
**Outcome: Implementation of ACEs screenings and strategy activities with fidelity and documenting adaptations**				
**Agent**	**Performance objectives**	**Determinants**	**Change methods**	**Practical applications and materials**
Medical Assistants	PO1. Attend ACEs screening and research procedures training PO2. Follow procedures before,	Having a working relationship with providers Time Proximity to patients / data Training	Skill building and guided practice Information transfer	Online videos Training manual and in-person orientation Trained coaches
	during and after screenings PO3. Document to submit billing for state reimbursement			
Community Health Advisors	PO1. Communicate with Medical Assistants and sub on screenings when clinic is short of personnel PO2. Provide service and educational resources to caregivers as part of follow up after screenings	Training	Modeling to ACEs screeners	Weekly updated excel database created for these screenings with local resources for mental health/behavioral referrals and waiting times Resource sheets for caregivers
		Expertise		
		Trust from caregivers/patients		
		Confidence on the care team's ability to support families after the ACEs screenings are completed and to address their needs		
Clinic Managers / DPP Co-lead	PO1. Identify eligible children every week	Perceived benefits of ACEs screenings for patients PO2. Supervise weekly completion of screenings PO3. Emphasize clinics' procedures already in place to address mandatory reporting and risk management with patients, and as part of the ACEs screenings	Supervisor audit and monitoring Information transfer and skill building	Academic partners presenting at the clinics' staff meetings Clinic managers included in planning meetings and ongoing coaching site visits ACEs written manual and training of care team
		Confidence on the care team's ability to support families after the ACEs screenings are completed and to address their needs		
**Sustainment phase**				
**Outcome: ACEs screenings and strategy activities are scaled up to other clinics at the healthcare system and they become part of primary care visit practices**				
**Agent**	**Performance objectives**	**Determinants**	**Change methods**	**Practical applications and materials**
Leadership	PO1. Distribute study results within the healthcare system, board of directors and state	Authority Outcome expectations	Increased commitment through results data	Short study results shared with leadership and scientific community
Training Department	PO2. Observe ACEs screenings trainings conducted in 3 of the five clinics PO2. Lead ACEs screenings trainings in the last two clinics P03. Lead ACEs screenings trainings in future clinics	Training Expertise	Facilitation through templates and procedures	Include ACEs screenings training materials in the healthcare system website

Leadership: Behavioral Health Department Chair, Pediatrics Chair, Chief Clinical Officer.

The lens of identifying determinants at the outer context during the planning group process also allowed the groups to identify the impact of new challenges that emerged during this phase of the process. One of those new challenges include the state of California, as a result of the COVID-19 pandemic, instituting a new policy requiring healthcare workers to show proof of vaccination by 7 October 2021 to remain in their jobs. As a result, our clinical partner lost clinic personnel (including Medical Assistants who were tasked with leading the ACEs screenings), and the project's timeline for the implementation phase had to be postponed.

For Task 5 [i.e., evaluate implementation outcomes; (26)] we will use mixed methods (e.g., REDCap, electronic health records, surveys, and interviews) to evaluate implementation outcomes by using a hybrid type 2, stepped-wedge cluster randomized trial design to test whether a multifaceted implementation strategy has a positive impact on fidelity, reach (i.e., proportion of eligible children screened for ACEs, and child level outcomes). Additional information on this IM Task 5 can be found elsewhere (34).

## Discussion

Through a seven-month IM collaborative process, researchers convened and collaborated with healthcare managers, clinic personnel, and caregivers of child patients to co-create implementation protocols through an IM process, guided by the EPIS framework. A need to identify and report implementation science engagement in research has been identified as a gap in the literature (36). We utilized a systematic planning approach to capacity building at the organizational and clinic levels and within a complex FQHC safety net healthcare system. The COVID-19 pandemic lengthened the IM process from the original plan of 5 months to 6/7 months due to staffing shortages and operational challenges at the clinics, which made scheduling frequent group meetings difficult. COVID-19 also made it harder for clinic staff to plan several months into the future, given the many uncertainties associated with the pandemic. In addition, the timeline for starting ACEs screenings had to be delayed due to lack of clinic personnel due to pandemic-related turnover. All meetings were conducted online and using audio and screen sharing only. Minor technical difficulties were common but not serious enough to impact the group process. Conversations with caregivers were held using cellphones, with two note-takers also participating.

We faced challenges during this process. A few stakeholders, mostly representing the Information Technology department, shared concerns about already having a plan in place; they had worries about their time and about not being part of the initial grant proposal conversations. The first author explained that having all stakeholders available for grant writing was not feasible and that the initial work was done with members of the research department and TIC workgroup at the FQHC. In addition, through IM, we were able to engage in a participatory process that helped develop the specific activities that were suitable for stakeholders and each clinic's workflow. This information seemed satisfactory for stakeholders to move forward. In addition, we held 15 follow-up meetings with smaller groups of stakeholders (e.g., care team members only); and separately with those with less perceived power (e.g., clinic staff and caregivers) to make those individuals feel safer and more comfortable in speaking directly.

Despite these challenges, including those posed by COVID-19 and its impact on the partner healthcare system and workforce, we were able to convene diverse groups of stakeholders and gather important information using a participatory approach. This approach increased buy-in among stakeholders. This support is reflected in the fact that the partner healthcare system reduced its collaborations with academic partners in 2020, and our study was one of only three studies approved to move forward despite the organizational stress brought about by the pandemic. Having a template of an implementation strategy to begin with was helpful to move the mapping process conversations along in a structured manner, while allowing changes on the strategy activities (forms) and preserving its goals (functions) (37). Lessons learned to engage stakeholders prior to the start of the funded study included establishing an academic-clinical partnership to work on relevant pro-bonus projects, creating a TIC workgroup comprised of academic partners and clinical personnel, including clinic champions in the grant budget to cover some of their time from day one of funding, and for clinical champions to share the background of the research behind ACEs through a monthly newsletter.

We acknowledge limitations in this study. The project started during the COVID-19 pandemic in May of 2021, which strained the FQHC system even more in terms of financial losses and workforce shortages. Related to this challenge, we relied on online meetings and clinic personnel often had technical difficulties accessing the meetings, and several of the stakeholders did not have video capacity. Despite these challenges, we were able to complete the IM planning process by having a flexible timeline, close communication within and across IM subgroups, and by having back up meeting times.

There are many commitment strategies that we have used with the most important that we used to overcome the obstacles and barriers related to ACEs screenings was linking with and supporting initiatives focused on trauma-informed care that can be used within health systems and practices. It is also important to understand that health systems are not static and if ACEs screenings as a routine practice in primary care settings are to be sustained, there should be sufficient attention to institutionalizing screenings, the incorporation of ACEs in the mission and vision of organizations as well as in the policies and procedures needed to communicate to all providers and staff that this is something that is expected, supported, and rewarded in the organization. It is also essential to increase the capacity of healthcare systems to link families to services as a result of these screenings, while addressing the limited capacity of local communities, especially rural and under-resourced areas (38), to absorb those referrals.

This study can inform other efforts, as projects seldom start from a blank slate. Often, there are implementation strategies already planned or discussed during the early phases of the implementation process. However, tailoring and adaptation are almost always needed, and collaboration can help to support and manage these processes (39). IM can be used as an evidence-informed approach for the exploration and preparation phases of the implementation process as a starting point for collaborative work with stakeholders. The goal of this process is to develop the protocols (who, how, why, when) and to tailor them to local clinic's workflows and procedures to increase the innovation's uptake. Mixed methods (REDCap, electronic health records, surveys and interviews) will be used to evaluate implementation outcomes by using a hybrid type 2, stepped-wedge cluster randomized trial design to test whether a multifaceted implementation strategy has a positive impact on fidelity, reach (i.e., proportion of eligible children screened for ACEs, and child-level outcomes).

Overall, the IM process that was informed by the EPIS framework facilitated consideration of outer system and inner organizational contexts as well as bridging factors that linked them. Our collaborative process allowed for a suitable approach for the inclusion of diverse stakeholders to co-engage in planning and pre-implementation of a complex health intervention. These interventions are delivered in dynamic and interdependent systems and require coordinated actions from multiple actors (40–42). For this study, the implementation of the ACEs screenings is immersed in a complex and dynamic outer state context related to the ACEs Aware screening policy, and to COVID-19 workplace requirements. In addition, the screenings require involvement of multiple individuals in a care team embedded within a clinic, which is in turn embedded in a large FQHC health system. However, inner context processes were the focus of much of the IM activities. For example, the community services representative person introduces the new health initiative to caregivers when they arrive at the clinic; medical assistants conduct the ACEs screenings; and pediatricians discuss the results of the screenings with families and make referrals to community services as needed. Then, referral service specialists follow up on those referrals with families to support engagement in services. One example of the benefits of stakeholder participation on these tasks was reflected in the fact that researchers observed higher buy-in and leadership from members of care teams and clinic managers who attended the IM sessions compared to those who were not part of the IM process. The former became champions within their own care teams and with their peers. In addition, the IM process allowed the research team to identify concerns among implementers and end-users related to health equity and unintended consequences of ACEs screenings and to set in place actions to address them early on during the preparation phase of EPIS. The focused IM process allowed the team to be more resilient to contextual changes and to be able to meet project milestones.

This study presented an example of how the team engaged diverse stakeholders across all IM Tasks. We also present how to integrate the IM process within a complex health system, while being guided by an implementation framework. The EPIS framework embodies process, determinants, and potential mechanisms in the implementation process. The synergy between IM and EPIS helped to frame conversations and discussions and to provide a conceptual starting point for this collaborative process. Integrating such an implementation theory with IM activities has the potential to advance implementation science while improving public health.

## Data availability statement

The original contributions presented in the study are included in the article/supplementary material, further inquiries can be directed to the corresponding author.

## Author contributions

MP is the principal investigator (PI) and had the main idea for this study. She was the lead for the IM process and meeting facilitator, wrote the first draft of this manuscript, and was responsible for all revisions. MF consulted with the PI on the use of the IM process in this project and attended IM sessions. GJ attended some of the IM sessions and coordinated the IM sub-groups' communication and logistics. JD attended an IM session and contributed to the literature review. GA has been involved with the project since conception, has supported the use of the EPIS framework in general and during the IM process and attended IM sessions. All authors have read, edited, and approved the final manuscript.

## Funding

This work was partially supported by the National Institute of Mental Health (R21MH123835).

## Conflict of interest

The authors declare that the research was conducted in the absence of any commercial or financial relationships that could be construed as a potential conflict of interest.

## Publisher's note

All claims expressed in this article are solely those of the authors and do not necessarily represent those of their affiliated organizations, or those of the publisher, the editors and the reviewers. Any product that may be evaluated in this article, or claim that may be made by its manufacturer, is not guaranteed or endorsed by the publisher.
